# Inflammation within the Abdominal Cavity

**Published:** 1870-03

**Authors:** F. K. Bailey

**Affiliations:** Knoxville, Tenn.


### CHICAGO MEDICAL EXAMINER.

N. S. DAVIS, M.D., EDITOR.

VOL. XI. MARCH, 1870. NO. 3.

### Original Contributions.

ARTICLE VIII.

### INFLAMMATION WITHIN THE ABDOMINAL CAVITY.

BY F. K. BAILEY, M.D., Knoxville, Tenn.

I read with some interest, in the EXAMINER for October, Dr. Breed's case of ileitis, as well as Dr. McElroy's remarks upon the same. I was reminded of a case which came once under my own observation; and the notes, made at the time, are here presented, as found in an old case book.

I was called, Saturday night, March 11th, 1848, to visit a lady 43 years old, mother of nine children, and seven living.

On the Tuesday preceding, she had walked more than a mile and back, causing much fatigue. The menses had missed for seven weeks, till a few days ago, when a slight flow of coagulum came on. Whether she was pregnant or not was not determined. The health had not been good for some time, having felt colic pains, which were accompanied with constipation. She was able to be about the house when I called, but the pulse was 120 or more. Head free from pain. Tongue white at the edges and base, but was brown through the centre.

Complains of pain in the right hypochondrium, with tenderness, upon pressure, over the whole abdominal region. In the course of the night, in the ascending colon there was a slight tumefaction. No action from the bowels for two or three days. Considerable nausea, with some vomiting. Under the impression that she had colic, various medicines, as suggested by the family, had been taken. With a view to allay pain and produce quiet, I gave opium in full doses, to be repeated every three or four hours, and applied a blister to the tumified spot. The extremities, which inclined to be cold, were to be kept warm.

*Sunday 12th, 9 A.M.* Found the stomach less irritable, but the pain and tenderness continued unrelieved.

Abdomen distended, and she complains of "wind in the stomach," as the principal item in her list of unpleasant feelings; and thinks if it could be thrown off, she would be better.

Ordered a dose of castor oil, and left with the understanding that I should visit her again at night. During the day, was informed that she was better, and concluded that the laxative had acted upon the bowels and removed the cause of trouble. I heard nothing more from her till Tuesday morning, when I was recalled.

Found the abdomen still distended and tender. There had been no alvine evacuation at all, but she had been able to sit up more or less during the two days previous. When I examined the case critically, there was no doubt that disease, in a stealthy manner, had steadily progressed, without any due appreciation of its being serious in its character, on the part of patient or the family, from Sunday till I called on Tuesday. There was little or no pain complained of, nor was it necessary to draw up the limbs in bed. The countenance was extremely anxious. Pulse feeble, very soft, and beating 160 times per minute. Occasional stercoraceous vomiting. Gave brandy freely, with hot applications to the extremities. Examined thoroughly to ascertain if there might not be strangulated hernia. Nothing of the kind had ever been noticed.

At noon, met Dr. Tillson, in consultation, and everything which seemed indicated was done to produce relief.

Vomiting became incessant, and the extremities were icy cold and of a deep livid appearance.

At four o'clock, the pulse became imperceptible. Vomiting constant, with eructations of gas. No pain at all, but incessant in asking for something to enable her to throw off the wind from the stomach.

I remained at the bedside during the entire night, and Wednesday morning only showed an aggravation of the morbid phenomena.

The extremities began to be of a purplish-red color, leaving a whiteness on pressure upon the skin.

Still no pain at all, though the whole abdomen had become enormously distended. The intellect remained perfectly clear, and there was absolute insomnia.

During the day, on Wednesday, the strength totally failed, or at least there was less inclination to move. No pulsation below the axilla, but the voice was clear, and she conversed freely with her numerous friends, till breathing ceased, at 11 o'clock P.M., and fully 30 hours after pulsation had ceased at the wrist.

*Post mortem* examination, 12 hours after death. Abdomen very much distended and exceedingly hard. Surface livid, particularly upon each side. On removing the anterior parieties, the omentum was found thickened, and upon its surface were found spots of ulceration, with patches containing pus. The peritoneal coat of the small intestines was extensively inflamed, and had become sphacelated in spots. The whole cavity was filled with serum, in which pus was found floating. The smell was so intolerable that it was found impossible to examine the large intestines, but the disorganization was equally evident in the lower portion of the cavity. The stomach was filled with flatus. The liver healthy.

What seemed remarkable, was the certainty that extensive disease had set in, while the patient could walk about and complain of but little pain. On Saturday night, when I first saw her, nothing but the frequent pulse indicated serious internal disease.

The only rational explanation of the uncommon features seen in this case, is the fact, that, at the time, there was an epidemic of pernicious intermittent prevalent. Numerous cases were then occurring of congestion of the brain. That same epidemic influence undoubtedly modified this case.*

From the fact that tumefaction appeared along the ascending colon, it is probable that the inflammation first commenced at that point. The occasional attacks to which she had been subject for some years were, perhaps, referrible to the same point.

In about a year after, I was called to see a man about 30, strong and robust, as a common laborer. He told me that he had just been eating very heartily of roast pig, after the usual day's work.

The abdomen rapidly became distended, and nausea and vomiting incessant, but without any pain. Pulse 140, very small and feeble. The condition above mentioned came on in a few hours after he ceased work. It was impossible to procure a motion from the bowels, and stercoraceous vomiting soon set in. This man lived about 48 hours after the first attack; and during the latter half of the time, he constantly begged for something to eat. Hunger was his chief complaint in his last hours, and he died asking for food. *Post mortem* revealed a gangrenous condition of the intestines, with serous effusion. Owing to the terrible fetor, a detailed examination was impracticable.

Besides, I have distinctly in mind three other cases similar to those described. Two were girls, 8 and 14 years of age, and one a young man. There was no *post mortem* in either of them, but the symptoms were such as to indicate congestion and gangrene. It seems to me that such cases may properly be denominated as *apoplexy* of the abdominal organs involved. Apoplexy literally signifies *intensity of seizure*, and, in this sense, it certainly is applicable.

I have neither time nor inclination to philosophize upon *causes*, either remote or immediate, which will produce such morbid manifestations.

It is natural for practitioners to report their cases which

* An account of the epidemic alluded to may be found in the Transactions of the Illinois State Medical Society, for 1857, under Congestive Intermittents.

result favorably, but I have given some which not only caused great anxiety in my own mind, but produced some excitement at the time in the community. Some writer, whom I can not now call to mind, describes an "acute phlegmonous enteritis."

In those cases above described, the term congestion and gangrenous might be properly applied.

*January 11th, 1870.*

